# The Aurora Kinase in *Trypanosoma brucei* Plays Distinctive Roles in Metaphase-Anaphase Transition and Cytokinetic Initiation

**DOI:** 10.1371/journal.ppat.1000575

**Published:** 2009-09-11

**Authors:** Ziyin Li, Takashi Umeyama, C. C. Wang

**Affiliations:** Department of Pharmaceutical Chemistry, University of California, San Francisco, California, United States of America; Yale University, United States of America

## Abstract

Aurora B kinase is an essential regulator of chromosome segregation with the action well characterized in eukaryotes. It is also implicated in cytokinesis, but the detailed mechanism remains less clear, partly due to the difficulty in separating the latter from the former function in a growing cell. A chemical genetic approach with an inhibitor of the enzyme added to a synchronized cell population at different stages of the cell cycle would probably solve this problem. In the deeply branched parasitic protozoan *Trypanosoma brucei*, an Aurora B homolog, TbAUK1, was found to control both chromosome segregation and cytokinetic initiation by evidence from RNAi and dominant negative mutation. To clearly separate these two functions, VX-680, an inhibitor of TbAUK1, was added to a synchronized *T. brucei* procyclic cell population at different cell cycle stages. The unique trans-localization pattern of the chromosomal passenger complex (CPC), consisting of TbAUK1 and two novel proteins TbCPC1 and TbCPC2, was monitored during mitosis and cytokinesis by following the migration of the proteins tagged with enhanced yellow fluorescence protein in live cells with time-lapse video microscopy. Inhibition of TbAUK1 function in S-phase, prophase or metaphase invariably arrests the cells in the metaphase, suggesting an action of TbAUK1 in promoting metaphase-anaphase transition. TbAUK1 inhibition in anaphase does not affect mitotic exit, but prevents trans-localization of the CPC from the spindle midzone to the anterior tip of the new flagellum attachment zone for cytokinetic initiation. The CPC in the midzone is dispersed back to the two segregated nuclei, while cytokinesis is inhibited. In and beyond telophase, TbAUK1 inhibition has no effect on the progression of cytokinesis or the subsequent G1, S and G2 phases until a new metaphase is attained. There are thus two clearly distinct points of TbAUK1 action in *T. brucei*: the metaphase-anaphase transition and cytokinetic initiation. This is the first time to our knowledge that the dual functions of an Aurora B homolog is dissected and separated into two clearly distinct time frames in a cell cycle.

## Author Summary

The chromosomal passenger complex (CPC) is essential for chromosome segregation and cytokinesis in eukaryotes, but the detailed mechanism of cytokinetic regulation remains less clear, partly due to the difficulty in separating the two functions in a growing cell. A chemical genetic approach by adding an inhibitor of the Aurora kinase in the CPC to a synchronized cell population at different cell cycle stages would probably solve this problem. The CPC in *Trypanosoma brucei* consists of an Aurora-like kinase (TbAUK1) and two novel proteins and bears little resemblance to the CPC in other eukaryotes. It moves from kinetochores to the spindle midzone during metaphase-anaphase transition, and then displays a unique trans-localization to the anterior end of the cell to initiate cytokinesis by moving from the anterior to the posterior end of the cell to separate it into two. To envision the role of TbAUK1 in driving this unusual process, we applied a chemical genetic approach and demonstrated that there are two distinct points of TbAUK1 action in *T. brucei*: the metaphase to anaphase transition and cytokinetic initiation. This is the first time to our knowledge that the dual functions of an Aurora B homolog is dissected and separated into two clearly distinct time frames in a cell cycle.

## Introduction

The Aurora-like kinases are essential mitotic regulators among eukaryotes. In metazoa, there are three such enzymes; Aurora A regulating spindle assembly, Aurora B promoting chromosome segregation and cytokinesis and Aurora C controlling chromosome segregation during male meiosis. But only a single Aurora-like kinase is required in budding and fission yeasts for spindle assembly and chromosome segregation without an apparent involvement in cytokinesis (for a review, see [1]).

Aurora B kinase in metazoa forms a chromosomal passenger complex (CPC) with three non-enzymatic partners, the inner centromere protein INCENP, Survivin, and Borealin [2]–[4]. In budding yeast, the single Aurora-like kinase Ipl1p also forms a CPC with three homologs of INCENP (Sli15p), Survivin (Bir1p) and Borealin (Nbl1p) [5],[6]. The mechanisms of CPC in detecting and correcting aberrant kinetochore-microtubule attachments during mitosis have been well characterized in yeast and metazoa. These involve the phosphorylation of several key kinetochore components by Aurora B [7]–[11], the activation of spindle checkpoint by targeting the checkpoint components to kinetochores [12] and the promotion of the association of BUBR1 with the anaphase-promoting complex/cyclosome (APC/C) [13]. The components of the kinetochore and the spindle checkpoint as well as the regulatory pathways governing kinetochore-microtubule attachments and chromosome segregation are well conserved from yeast to human.

The potential role of Aurora B in promoting cytokinesis in metazoa has, however, not yet been clearly delineated or well separated from its regulatory function on mitosis (for a review, see [1]). An Aurora B-mediated phosphorylation of the two subunits in the centraspindlin complex, the Rho GTPase activating protein MgcRacGAP/RacGAP50C/CYK-4 and the kinesin MKLP1/Pavarotti/ZEN-4, is essential for targeting the centraspindlin complex to the spindle midzone where it binds Ect2, a guanine nucleotide exchange factor (GEF) [14]–[16]. Ect2 then activates the small GTPase RhoA in the equatorial region of the cell membrane to promote the formation of the actomyosin contractile ring that constitutes the initial cleavage furrow. The latter then closes onto the midzone to complete the process of cytokinesis [17]. Recently, a quantitative analysis of Aurora B phosphorylation dynamics indicated the formation of a spatial phosphorylation gradient along the division axis early in the anaphase in HeLa cells [18]. This gradient was postulated to provide the spatial information for positioning the actomyosin ring. But the range of potential substrates of Aurora B and the specific time frame of Aurora B action required for cytokinesis remain unclear. Nor is it well understood whether the Aurora B action following the metaphase-anaphase transition plays an essential role in promoting mitotic exit, cytokinetic initiation, or cytokinetic completion.

In *Trypanosoma brucei*, a parasitc protazoan that causes human sleeping sickness in Sub-Saharan Africa, a single functional Aurora-like kinase, TbAUK1, is responsible for promoting spindle assembly, chromosome segregation as well as cytokinesis [19],[20]. Homologs of INCENP, Borealin and Survivin, however, have not been found in the trypanosome genome [21]. Instead, a novel CPC consisting of TbAUK1 and two novel proteins TbCPC1 and TbCPC2, which bear no structural similarity to those three non-enzymatic proteins, was identified in *T. brucei* [22]. This CPC displays a typical sub-cellular localization pattern during mitosis similar to that of the metazoan CPC [22]. It associates with the chromosomes during G2 phase, with kinetochores in metaphase, and then moves to the spindle midzone in anaphase.

The trypanosomes are known to divide by a pattern totally different from that of metazoa and yeast. They divide longitudinally from the anterior toward the posterior end of the cell [23]. A most unusual pattern of CPC trans-localization has since been observed in trypanosomes toward the end of mitosis and beginning of cytokinesis [22],[24]. The mid-portion of the elongated spindle bearing the midzone starts to bend toward the dorsal side of the cell, where the flagellum attachment zone (FAZ) is aligned. The CPC in the midzone is then transferred to the mid-point of the cellular dorsal side, and then moves to the anterior end of the cell to initiate cytokinesis by moving from the anterior toward the posterior end accompanied with a division of the cell into two [22],[24]. This unusual mode of cell division in *T. brucei*, apparently mediated by the CPC, indicates a unique mechanism of cytokinesis that could be shared by all the flagellates that divide longitudinally. One of the burning questions from this observation is whether the TbAUK1 function in the CPC plays an essential role in CPC trans-localization and cytokinesis. A previous RNAi depletion of TbAUK1 from an asynchronous trypanosome population, which was mostly in the G1 phase, arrested the cells in G2/M phase and enriched cells with an enlarged nucleus and two widely separated kinetoplasts [19], indicating that both mitosis and cytokinetic initiation are blocked. In a separate study, an over-expression of an inactive TbAUK1-K58R mutant in *T. brucei* exerted a dominant-negative effect resulting in a virtually identical outcome like that from the RNAi experiment [20]. It is thus highly likely that TbAUK1 has also a dual function in *T. brucei* in regulating metaphase-anaphase transition and cytokinetic initiation. The genetic studies have their limitations in leading the asynchronous cells, which are mostly in the G1 phase, to a phenotype defective in metaphase-anaphase transition thus masking the next potential role of TbAUK1 in controlling cytokinesis [22]. Furthermore, RNAi-mediated silencing of any one of the three CPC subunits led invariably to a disintegration of the complex, making it difficult to study the potential role of TbAUK1 within the CPC complex during cell cycle progression [22].

Here we applied a chemical genetic approach through inhibiting TbAUK1 kinase activity with a small-molecule inhibitor, VX-680, at different cell cycle stages in a synchronized cell population. It allowed us to dissect the two functions of TbAUK1 at different stages of the cell cycle, which could not be accomplished by genetic manipulations. VX-680 was originally discovered as a selective inhibitor against human Aurora kinases, and has apparent IC_50_ values of 0.6, 18 and 4.6 nM against human Aurora A, B, and C, respectively [25]. It showed greater than 100-fold selectivity for Aurora kinases over 55 other kinases except for Fms-related tyrosine kinase-3, which has been found missing from *T. brucei* [26],[27]. We found in this inhibitor an IC_50_ value of 190 nM against TbAUK1. It arrested an unsynchronized trypanosome cell proliferation in the G2/M phase with an enlarged nucleus and two segregated kinetoplasts in each cell as having been observed from an RNAi knockdown of TbAUK1 [19] or an over-expressed TbAUK1-K58R negative mutant [20]. By adding VX-680 to a synchronized trypanosome cell population at different stages of the cell cycle, we were able to uncover what was not found in an RNAi experiment. We demonstrated that TbAUK1 plays essential roles at two distinctive stages of the cell cycle; (1) the metaphase-anaphase transition; (2) the trans-localization of the CPC from the spindle midzone to the dorsal anterior of the cell at the beginning of cytokinesis. The dual roles of an Aurora-like kinase have thus been clearly dissected in this model organism (see Discussion).

## Results

### VX-680 inhibits TbAUK1 kinase activity and cell cycle progression in *T. brucei*

**Figure 1:**
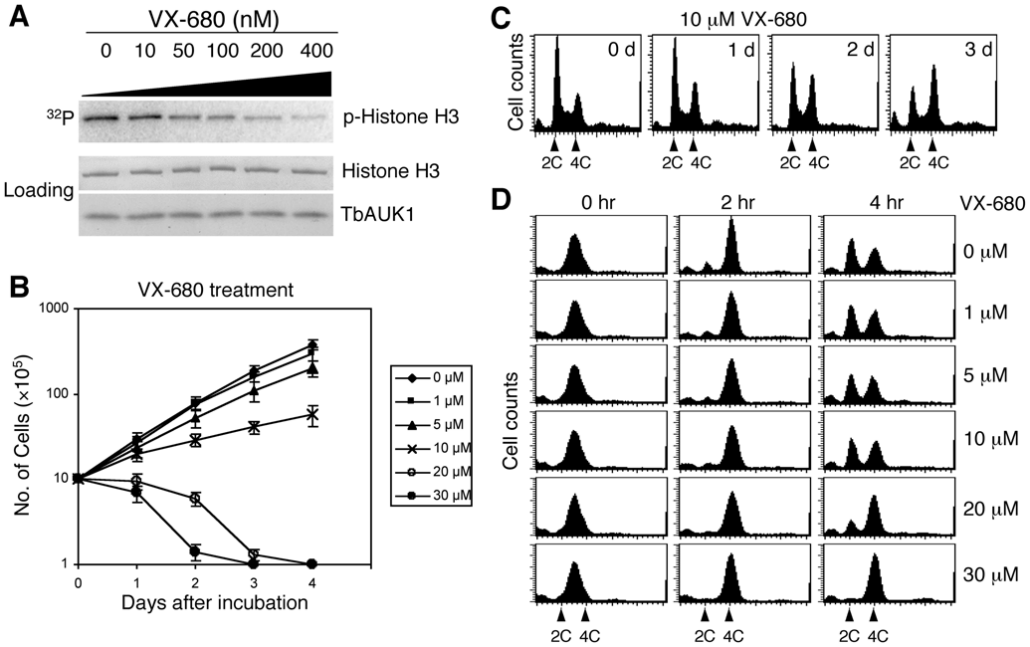
Effect of VX-680 on TbAUK1 kinase activity, cell growth and cell cycle progression in the procyclic trypanosomes. (A). Effect of VX-680 on TbAUK1 kinase activity. Recombinant TbAUK1 and histone H3 each fused with GST were purified and incubated with different concentrations of VX-680 (shown at the top) in the presence of γ-^32^P-ATP. The incubation was carried out at room temperature for 60 min. The assay mixtures were separated in SDS-PAGE and analyzed by Phosphor-Imager for the extent of histone H3 phosphorylation. (B). Effect of VX-680 on cell proliferation. Procyclic 427 cell line was cultivated *in vitro* in the presence of different concentrations of VX-680. Cell density was counted daily and plotted against incubation time. The results were from three independent experiments. (C). Effect of VX-680 on cell cycle progression in a non-synchronous trypanosome cell population. Procyclic 427 cell line was incubated with 10 µM VX-680 for 3 days. Cells were harvested each day after VX-680 treatment for flow cytometry analysis. (D). Effect of VX-680 on cell cycle progression in synchronized trypanosome cells. Procyclic 427 cell line was synchronized in late S-phase with 0.3 mM hydroxyurea for 16 hrs and released. Cells were then incubated with different concentrations of VX-680 for 2 and 4 hrs, respectively and subjected to flow cytometry analysis.

To investigate the potential inhibitory effect of VX-680 on TbAUK1 kinase activity, recombinant GST (glutathione-S-transferase)-tagged TbAUK1 and histone H3 were expressed in transformed *Escherichia coli*, purified to near homogeneity and used for *in vitro* assay of the TbAUK1 kinase activity [20]. VX-680 was dissolved in DMSO and added to the assay mixture at a final concentration of 0, 10, 50, 100, 200 or 400 nM, respectively. TbAUK1 phosphorylated histone H3 with a strong signal in the no drug control, but the activity became increasingly inhibited at higher concentrations of VX-680 (Fig. 1A). The IC_50_ value of VX-680 against TbAUK1 was calculated to be 190 nM.

The potential effect of VX-680 on trypanosome *in vitro* proliferation was then investigated. Procyclic cells were incubated with VX-680 ranging from 1 to 30 µM, and the cell growth was monitored at different time intervals. The cells grew with a slightly slower rate than the control in the presence of 1 or 5 µM VX-680, but were significantly slowed down to half of the control rate at 10 µM VX-680 (Fig. 1B). At 20 µM and 30 µM, VX-680 totally inhibited cell growth, which led to an eventual cell death after 2 to 3 days (Fig. 1B). The IC_50_ value of VX-680 on trypanosome cell growth was estimated to be 10 µM.

To test the effect of VX-680 on cell cycle progression, unsynchronized procyclic cells were treated with 10 µM VX-680 and subjected to daily flow cytometry analysis (Fig. 1C) and examination for numbers of nuclei and kinetoplasts in each cell (data not shown). The outcome turned out to be similar to that from a TbAUK1 RNAi experiment [19]. There was an enrichment of cells with 4C DNA content (G2/M cells) and a corresponding increase of cells with an enlarged nucleus and two segregated kinetoplasts (1N*2K). Apparently, VX-680 treatment and TbAUK1 RNAi of the procyclic cells result in the same phenotype, suggesting that VX-680 acts by primarily inhibiting TbAUK1 in the current experimental setting.

In a separate experiment, the procyclic cells were synchronized by hydroxyurea using a procedure slightly modified from the previously published method [28] and released in late S-phase. VX-680 was then added to the cells at various concentrations ranging from 1 to 30 µM at the time of release, and the cells were analyzed by flow cytometry 2 and 4 hrs thereafter. In the no-drug control, the synchronized cells proceeded from S-phase to G2/M phase within 2 hrs and then progressed to cell division to produce G1 cells after 4 hrs (Fig. 1D). In the presence of 1 µM, 5 µM or 10 µM VX-680, there was little inhibition of the cell cycle progression within the first 4 hrs (Fig. 1D). But 20 µM VX-680 enhanced the G2/M cells to over 80% of the total population after 4 hrs, whereas 30 µM VX-680 accumulated G2/M cell to over 95% of the total population without cell division after 4 hrs of treatment (Fig. 1D). Thus, within a much shortened time span of 2 hours, comparing with the 24 hr period required in the TbAUK1 RNAi study [19], an application of VX-680 at a concentration of 30 µM to the synchronized cells in late S-phase resulted in an essentially total arrest of the cells in G2/M phase. This 30 µM concentration of VX-680 was thus chosen for the rest of the experiments, because of its near total arrest of the cells in the G2/M phase within a relatively short time

### VX-680 disrupts chromosome segregation when added to the trypanosomes within 2 hours after release from S phase

The procyclic cells expressing tagged TbCPC1-EYFP, TbCPC2-EYFP or TbAUK1-EYFP at the apparent endogenous levels [24] were synchronized by hydroxyurea and released in late S-phase. VX-680 was added to the cells at 0, 1, 2, 3, 4, 5, 6 and 7 hrs after the release. Hourly samples after the release were analyzed by flow cytometry and examined by fluorescence microscopy to localize the fluorescent proteins.

**Figure 2:**
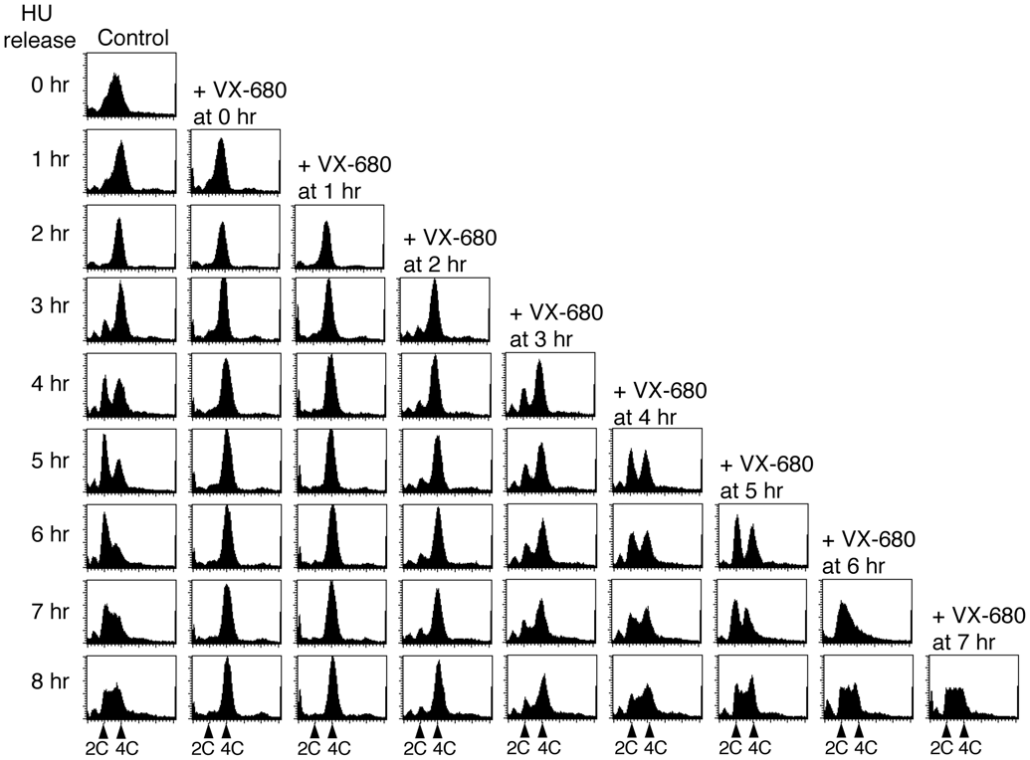
Flow cytometry analysis of cell cycle progression of hydroxyurea-synchronized *T. brucei* procyclic cells with VX-680 added at different time intervals after the release. Procyclic cells expressing endogenous EYFP-tagged TbCPC1 were synchronized to late S phase by incubating with 0.3 mM hydroxyurea for 16 hrs and released. Cells were cultivated in fresh media for 8 hrs with cell samples harvested every hour for flow cytometry analysis. VX-680 (30 µM) was added at 0, 1, 2, 3, 4, 5, 6, and 7 hrs after the release.

In the no drug control, the cells progressed synchronously and steadily from S-phase through G2 to mitosis within the first 2 hrs, and began to show the initial sign of cell division after 3 hrs by the emergence of a tiny G1 peak (with 2C DNA content) (Fig. 2). The G1 cells, likely representing the newly divided cells, increased rapidly between the 3^rd^ and the 5^th^ hour accompanied by a corresponding decrease of G2/M cells (with 4C DNA content). This was clearly a period of active cell division. The apparent increase of G1 cell population slowed down subsequently and stopped at the 6^th^ hour with a steadily increasing S-phase cell population, which kept increasing into the 7^th^ and 8^th^ hour accompanied with a steady decrease of G1 cells. The profile of cells at 8 hrs after the release resembled that at 0 hr except for some G1 cells apparently still remaining in the 8^th^ hour sample, suggesting that within a relatively short additional time, the cells would be mostly back to S phase, thus completing a well-synchronized cell cycle progression (Fig. 2).

When the cells were treated with VX-680 immediately after the release, they were able to proceed to the G2/M phase within the first 2 hours at a rate similar to that of the no drug control. It suggests that TbAUK1 plays no role in the S to G2/M transition in trypanosomes. The cells were, however, unable to proceed beyond the G2/M phase to cell-division as there were no G1 cells emerging thereafter. They remained arrested at the G2/M boundary for the rest of the incubation period up to 8 hrs with almost 98% of the cells possessing a 4C DNA content (Fig. 2 and Fig. S1). Similar results were obtained when VX-680 was added at 1 hr or 2 hrs after the release, suggesting that, after 2 hrs of progression from the late S phase, the cells reached a specific stage requiring TbAUK1 to play a crucial role to move on (Fig. 2 and Fig. S1). When the drug was added 3 hours after the release, cell division was just initiated at that time (see the no-drug control in Fig. 2). The drug first slowed down the emergence of G1 cells, then stopped it totally and eventually reduced the number of G1 cells as the S-phase cells started to increase gradually (Fig. 2 and Fig. S1). This particular 3 hr time point appears to be a crucial moment when TbAUK1 plays an apparently critical function in promoting cytokinetic initiation beyond the G2/M phase, because when VX-680 was added to the cells at 4, 5, 6 and 7 hrs after the release, there was little apparent disturbing effect on the subsequent cell cycle progression (Fig. 2 and Fig. S1). Cytokinesis was thus apparently already initiated among most of the cells after 4 hrs of cell cycle progression from the late S phase. Further progressions through cytokinesis, the next G1 phase, the S phase and the G2 phase apparently do not require the function of TbAUK1.

**Figure 3:**
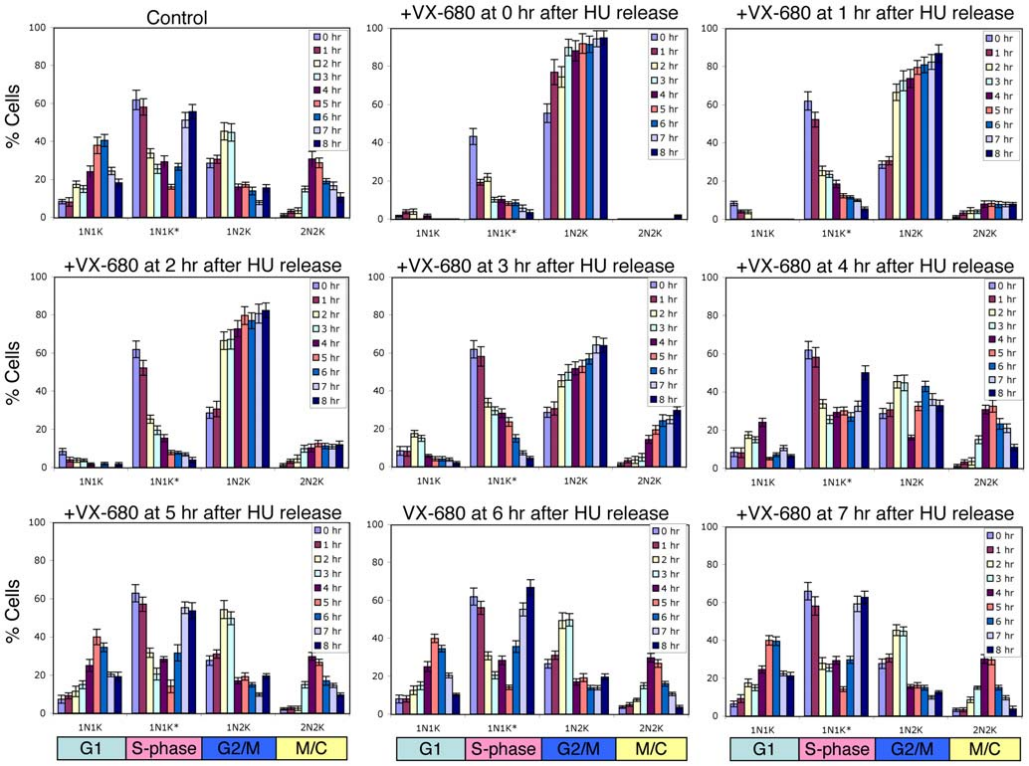
Quantification of different cell types after VX-680 treatment of hydroxyurea-synchronized *T. brucei* procyclic cells. Cells treated with VX-680 at different time intervals after the release from hydroxyurea-treatment were collected, fixed with paraformaldehyde, stained with DAPI and the number of cells with different numbers of nuclei and kinetoplasts were counted. Data were presented as the mean percentage ±S.D. of total cells counted (>200) from three independent experiments.

These synchronized cells, treated with VX-680 at different stages of their cell cycle progression, were also stained with DAPI for the numbers and sizes of nucleus (N) and kinetoplasts (K) in individual cells. In the no drug control, the cells consisting of one nucleus and one elongated kinetoplast (1N1K*), which represent cells in S-phase [29], were in the majority at the beginning (Fig. 3). They rapidly vanished within the first 5 hrs after release but re-emerged between the 6^th^ to 8^th^ hours. This initial decrease in 1N1K* cells was closely followed by an increase in 1N2K cells and then 2N2K cells and finally the 1N1K cells, which are apparently in the G1 phase. The time course of changes in numbers of nucleus and kinetoplast overlaps well with the anticipated cell cycle progression in trypanosomes [30]. Kinetoplast segregation is known to precede the nuclear division, whereas the transition from 2N2K cells to 1N1K cells represents cell division (Fig. 3).

When VX-680 was added to the synchronized cells at 0 to 2 hrs after the release from the late S-phase, there was a declining 1N1K* population accompanied by a rapidly increasing population of 1N2K cells as observed in the control. But the latter were apparently not further converted to 2N2K or 1N1K cells (Fig. 3). They are thus likely arrested in the G2/M phase unable to proceed into nuclear division or cytokinesis. This outcome is similar to that observed from knocking down TbAUK1 with RNAi over a longer time span of 24 hours [19]. The slowness in developing a comparable phenotype from an RNAi experiment could be attributed to the time required for degradation of mRNA and protein, which was not encountered in an enzyme inhibition study.

After the release from the late S-phase for 3 hrs, the drug addition enhanced the 2N2K cell population (Fig. 3), but the subsequent increase in 1N1K cells was much reduced, indicating that cytokinesis was inhibited. When VX-680 was added after the cell was released for 4, 5, 6, or 7 hours, however, the 2N2K cells that accumulated during the initial 4 to 5 hours declined and were accompanied with a corresponding increase of 1N1K cells similar to that in the no-drug control (Fig. 3). The TbAUK1 function is thus apparently no longer required for cell cycle progression once the cells have passed the point of cytokinetic initiation.

These experiments provided an important indication that TbAUK1 plays critical roles in two separate stages of the cell cycle in trypanosomes; the completion of mitosis and the initiation of cytokinesis.

### Early VX-680 inhibition of TbAUK1 prevents trans-localization of the CPC from the metaphase plate to the spindle midzone in anaphase

**Figure 4:**
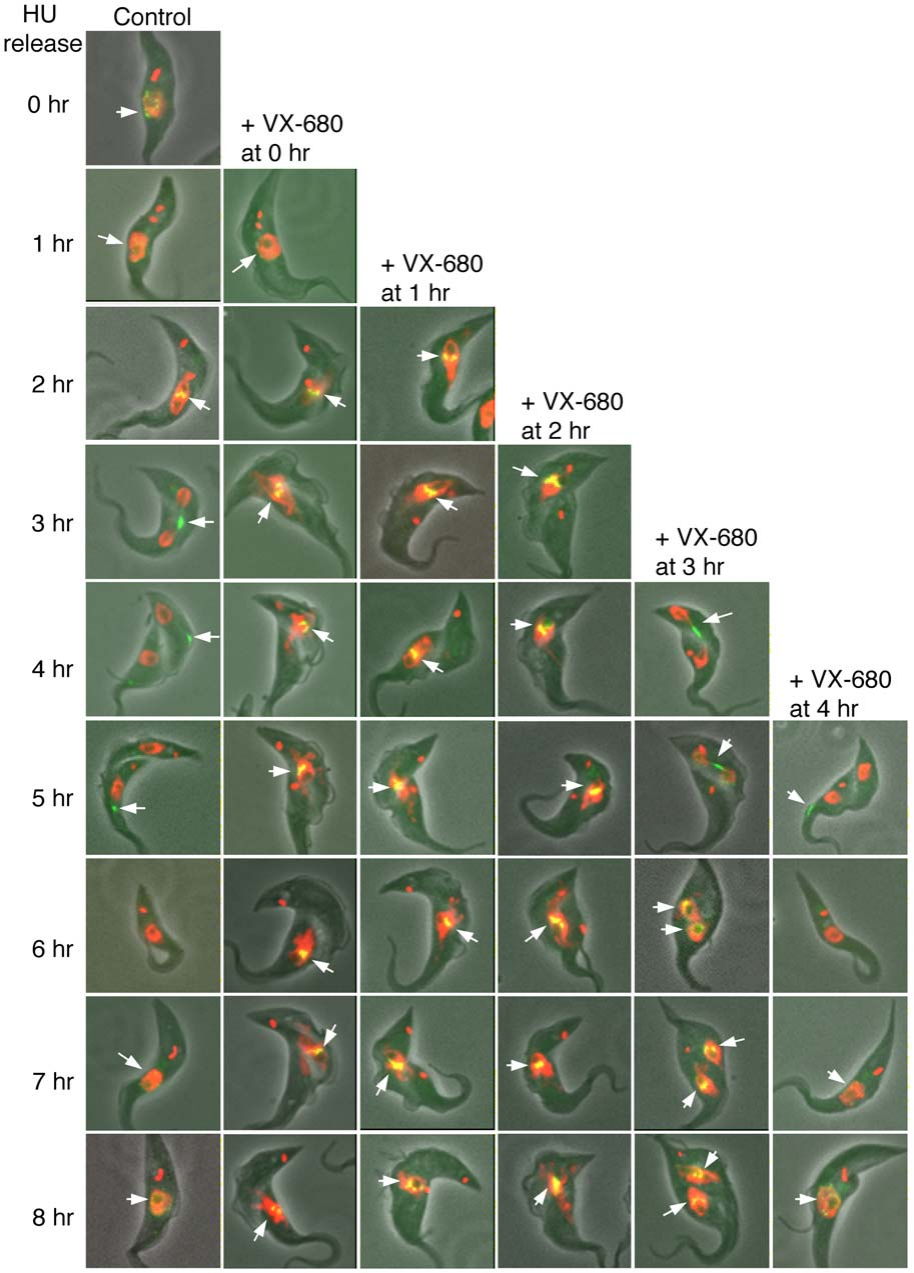
Effects of VX-680 on trans-localization of TbCPC1-EYFP in hydroxyurea-synchronized *T. brucei* procyclic cells. Procyclic cells expressing the TbCPC1-EYFP fusion protein were synchronized with 0.3 mM hydroxyurea for 16 hours and released. They were treated with 30 µM VX-680 after having been released for 0, 1, 2, 3, 4, 5, 6 or 7 hrs. Cell samples were harvested every hour after the release, fixed with 4% paraformaldehyde, stained with DAPI, and examined with a fluorescence microscope. The data shown are merged images of phase contrast (gray), DAPI-stained DNA (red), and TbCPC1-EYFP (green). The arrows point to the TbCPC1-EYFP signal. The same images not merged together are presented individually in Figures S2, S3, S4, S5, S6, S7 for enhanced clarity.

To investigate the potential effect of TbAUK1 inactivation on the trans-localization of the CPC during cell cycle progression, the synchronized procyclic trypanosome cells expressing tagged TbCPC1-EYFP at the apparent endogenous level were released in the late S-phase and treated with VX-680 at different time points thereafter. The localization of TbCPC1-EYFP was then examined with a fluorescence microscope. In the control cells, TbCPC1-EYFP was dispersed in the nucleus after the release for 1 hr but became concentrated on the metaphase plate after 2 hrs (Fig. 4; arrow). It was then trans-localized to the spindle midzone between the two segregating nuclei 3 hrs after the release and became further enriched in the midzone (Fig. 4; arrow). The protein then moved toward the dorsal mid-point of the cell and migrated toward the anterior end 4 hrs after the release (Fig. 4; arrow), and was eventually and exclusively located at the anterior end of the cell at the 5^th^ hr (Fig. 4; arrow). The migration of TbCPC1-EYFP during the process of cytokinesis was difficult to monitor because it had a very short duration and could be captured only by time-lapse video microscopy (see below). When the cells entered the next round of cell cycle, TbCPC1-EYFP was no longer visible in the G1 phase at the 6^th^ hr of release, but re-emerged in the nucleus when the cell entered S phase again after 7 to 8 hrs (Fig. 4). This dynamic pattern of trans-localization of the EYFP-tagged TbCPC1 agrees with that of the HA-tagged TbAUK1, TbCPC1 and TbCPC2 in both the procyclic and bloodstream forms of *T. brucei* observed previously [22],[24]. A time-lapse video microscopy was also employed to record this event in the current study (Video S1). We started the time-lapse experiment when TbCPC1-EYFP was already localized to the spindle midzone. The protein then moved to the dorsal mid-point of the cell between the 11th and 17th min. A portion of the fluorescent spot then quickly appeared at the anterior end of the cell at the 20th min, which was followed by a movement of the entire TbCPC1-EYFP spot to the anterior end within 56 minutes. A dramatic sliding action of TbCPC1-EYFP along the apparent cleavage ingression from the anterior to the posterior end of the cell then occurred from the 104th to the 140th min resulting in dividing the cell (Video S1).

When VX-680 was added to the cells between 0 to 2 hrs after the release, TbCPC1-EYFP became concentrated on the metaphase plate after 2 hrs as in the control cell regardless of the precise time of drug addition (Fig. 4; arrows). TbCPC1-EYFP then remained on the metaphase plate for the rest of the experimental period, indicating that TbAUK1 plays an essential role in promoting the transition from metaphase to anaphase. This observation was further confirmed by data from time-lapse video microscopy (Video S2). A cell arrested in the metaphase with TbCPC2-EYFP localized to the metaphase plate from a synchronized cell population treated with VX-680 at time 0 of their release was identified after 2 hrs (Video S2). The cell was monitored continuously for the next 6 hrs. But there was no sign of either progression beyond the metaphase or dissociation of TbCPC2-EYFP from the metaphase plate during this prolonged incubation. Similar results were also obtained with the cells expressing EYFP-tagged TbAUK1 (data not shown).

### VX-680 inhibition of TbAUK1 suggests a disrupted chromosome alignment on the metaphase plate

**Figure 5:**
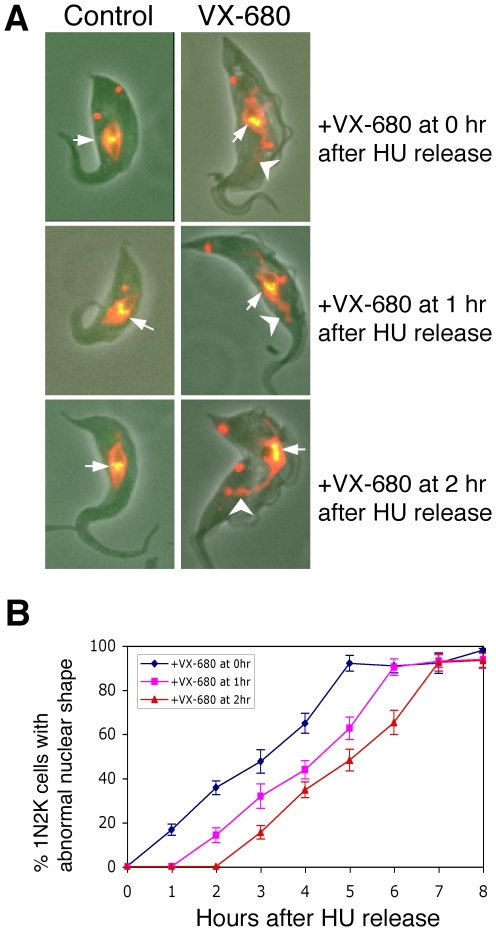
Effect of VX-680 treatment on the size and shape of nucleus. *T. brucei* procyclic cells expressing the TbCPC1-EYFP fusion protein were synchronized with 0.3 mM hydroxyurea and released. Cells were treated with 30 µM VX-680 after having been released for 0, 1, or 2 hrs and the incubation was continued until the 8^th^ hour after release. Cell samples were fixed in paraformaldehyde, stained with DAPI and examined with a fluorescence microscope. (A). The control and VX-680-treated cells in metaphase with TbCPC1-EYFP concentrated on the metaphase plate. The merged images are derived from phase contrast (gray), DAPI-stained DNA (red), and TbCPC1-EYFP (green). The arrows point to the TbCPC1-EYFP signal and arrowheads point to the irregular shapes of DNA stain. The same images not merged together are presented in Figure S8 for increased clarity; (B). Percentages of 1N2K cells with the apparently mis-aligned chromosomes in VX-680 treated cells.

Aurora B kinase in the CPC is known to adjust chromosome bi-orientation for proper kinetochore-microtubule attachments during metaphase in eukaryotes [4], [31]–[34]. This is achieved through Aurora B-mediated phosphorylation of the kinetochore components and activation of the spindle assembly checkpoint to prevent premature progression into the anaphase (for a review, see [1]). In trypanosomes, the chromatin does not condense and chromosome alignment cannot be clearly distinguished by DAPI staining. However, when VX-680 was added to the cells prior to the onset of metaphase and incubated up to 8 hrs thereafter, essentially all the cells were found arrested in metaphase with TbCPC1-EYFP remaining on the metaphase plate. But the shape of the DAPI-stained nucleus became irregular and enlarged, which could suggest mis-aligned chromosomes (Fig. 5A; arrowheads). This is in contrast to the control metaphase cells in which the DAPI-stained nucleus was in a typical regular diamond shape (Fig. 5A; see also [35]). The irregular and enlarged nucleus was observed among approximately 20% of the cells 1 hr after VX-680 addition and progressed beyond 95% after 8 hrs (Fig. 5B). The TbAUK1 kinase activity could be thus required for proper chromosome alignment on the metaphase plate.

### VX-680 inhibition of TbAUK1 after the completion of metaphase-anaphase transition abolishes CPC trans-localization from the spindle midzone to the cleavage furrow

To investigate the potential effect of TbAUK1 inactivation on the continuous CPC trans-localization after it has already reached the spindle midzone, we added VX-680 to the synchronized cells 3 hrs after the release, while the majority of cells has entered the anaphase with TbCPC1-EYFP enriched in the spindle midzone (Fig. 4; arrow). TbCPC1-EYFP remained in the midzone for 1 to 2 hrs after the drug treatment without any sign of movement to the dorsal mid-point of the cell, while nuclear division proceeded at a rate similar to that of the no-drug control (Fig. 4; arrows). When nuclear division was completed, TbCPC1-EYFP was found evenly distributed in the two segregated nuclei and the bi-nucleated cells showed no sign of cell division upon prolonged incubation (Fig. 4; arrows in the panel of +VX-680 at 3 hr). Apparently, VX-680 treatment of cells in the anaphase blocked further trans-localization of TbCPC1-EYFP from the spindle midzone and inhibited cytokinetic initiation, but did not hinder the completion of mitosis. TbAUK1 is thus likely required for CPC trans-localization from the spindle midzone to the cell dorsal mid-point to initiate cytokinesis. To further confirm this finding, cells treated with VX-680 3 hrs after release from late S phase were examined with time-lapse video microscopy. TbCPC2-EYFP disappeared from the spindle midzone after 30 min of the drug treatment but re-appeared in the two nuclei immediately thereafter, while no cell division took place (Video S3). It appears that, in the absence of a functioning TbAUK1, the CPC becomes dissociated from the spindle midzone and redistributed back into the two newly formed nuclei upon the completion of mitosis, while the cell remains undivided, suggesting that cytokinetic initiation was inhibited by TbAUK1 inactivation.

To test a possible involvement of TbAUK1 in driving trans-localization of the CPC from the dorsal mid-point of the cell to the anterior end, VX-680 was added to the cells 4 hrs after the release, when the cells have progressed to the telophase and TbCPC1-EYFP is partly on the dorsal mid-point of the cell and partly at the anterior end (Fig. 4; arrows). TbCPC1-EYFP was found capable of traveling from the dorsal mid-point to the anterior end of the cell followed by conversion of bi-nucleate to single nucleate cells indicating cell division (Fig. 4, panel +VX-680 at 4 hr). Similar results were also obtained with the cells expressing EYFP-fused TbAUK1 and TbCPC2 (data not shown). Thus, the TbAUK1 kinase function is no longer required once the CPC has been trans-localized to the dorsal mid-point. Cytokinesis can proceed to completion from that point onward without TbAUK1 activity.

## Discussion

In this report, we applied a chemical genetic approach to precisely dissect the specific roles of an Aurora-like kinase TbAUK1 in *T. brucei* in regulating the cell cycle progression and the trans-localization of the CPC. It enabled us to abolish TbAUK1 function at a specific given moment during the cell cycle progression of a synchronized cell population without disrupting the CPC structure [22],[24] and monitor the consequences thereafter. The outcome from the present study suggests that the observed drug effect on a synchronized *T. brucei* population within the initial hours of the cell cycle progression is essentially identical to that from knocking down TbAUK1 with RNAi [19] or from over-expressing TbAUK1-K58R negative mutant in trypanosome cells [20]. The drug effect we have observed in the present study could be thus attributed primarily to the inhibition of TbAUK1. Recently, Jetton et al. [36] tested another known inhibitor of Aurora B, Hesperadin, on the bloodstream form *T. brucei* cells and observed blocked nuclear division and cytokinesis but not other aspects of the cell cycle. It corroborates with our current finding.

TbAUK1 function is apparently not required for cell cycle progression from late S phase to the onset of metaphase. But continued inhibition of TbAUK1 up to the metaphase prevents the cells from proceeding further into anaphase. This finding agrees with the observed function of Aurora B in metazoa, in which the chromosome segregation defect caused by Aurora B deficiency attributes to defective bipolar spindle assembly [3],[32],[37] and improper kinetochore-microtubule attachments [4],[12],[31],[33],[38],[39]. These aberrant attachments activate the spindle assembly checkpoint, which in turn inhibits APC/C, resulting in metaphase arrest [40]. TbAUK1 is apparently performing a similar function during this particular phase of mitosis in *T. brucei*. DAPI-stained DNA patterns suggesting mis-aligned chromosomes are detected around the metaphase plate, which could be likely attributed to improper kinetochore-microtubule attachments when TbAUK1 is inhibited (Fig. 5).

Many well-conserved proteins are involved in regulating kinetochore-microtubule attachment in metazoa and yeast. But the majority of them do not find their structural homologs in the trypanosome genome [21], which raises an interesting question on how TbAUK1 regulates kinetochore-microtubule attachment and chromosome segregation in the absence of these crucial components. It is possible that the trypanosome kinetochores and spindle checkpoint are composed of structurally distinct proteins that could cooperate with TbAUK1 in fulfilling the roles of promoting faithful chromosome segregation. Future work will be directed to identify these proteins and to investigate their association with TbAUK1 in regulating chromosome segregation.

A few of the TbAUK1-associated proteins, other than TbCPC1 and TbCPC2, have been identified in *T. brucei* recently [22],[41]. The Tousled-like kinase TbTLK1, which is a substrate of TbAUK1 and is capable of auto-phosphorylating, was found co-immunoprecipitated with TbAUK1 but concentrated at the spindle poles during mitosis. It could play a role in regulating spindle assembly [41]. TbAUK1 is also associated with a novel kinesin-like protein TbKIN-A that co-localizes with the CPC on the chromosomes during prophase, and the spindle midzone in anaphase [22]. At the critical moment of nuclear division and cytokinetic initiation, however, TbKIN-A does not trans-localize with the CPC, but, instead, disperses from the spindle midzone and re-distributes back into the two newly formed nuclei [22]. TbKIN-A is thus probably playing roles only during the mitosis. Another kinesin-like protein, TbKIN-B, was found associated with TbAUK1, TbCPC1, TbCPC2, and TbTLK1 [22],[24]. It trans-localizes in the same pattern as that of TbKIN-A and is thus most likely involved only in regulating mitosis in trypanosomes.

The precise mechanism of CPC-mediated initiation of cytokinesis in *T. brucei* is not known. Since the cell divides from the anterior to the posterior end [23], a phosphorylation gradient generated by the Aurora B from the midzone as observed in metazoa [18] would thus not provide any useful spatial information for positioning cleavage furrow ingression in *T. brucei*, which is not constituted by an actomyosin ring in the first place [42]. Our current investigation has clearly indicated that TbAUK1 activity is required for trans-localization of the CPC from the midzone to the dorsal mid-point of the cell in late anaphase, presumably by crossing the nuclear envelope. Once this phase of trans-localization is accomplished, further migration of the CPC to the anterior tip followed by a rapid movement toward the posterior end for completion of cytokinesis are apparently no longer dependent on the activity of TbAUK1. The latter is thus specifically required only for the initial CPC trans-localization to start cytokinesis. When trypanosome cells have already reached anaphase but have not yet initiated cytokinesis, loss of TbAUK1 activity stops the trans-localization of the CPC, but mitosis proceeds to completion. The CPC is simply re-distributed to the two newly formed nuclei like TbKIN-A and TbKIN-B [22]. Detailed mechanisms in this remarkably unique action of TbAUK1 in initiating CPC trans-localization and cytokinesis will be the subject for much intensive investigation in the future.

In summary, we have succeeded in clearly demonstrating two discrete functions of TbAUK1 in *T. brucei* during its cell cycle progression. The essential role of TbAUK1 in promoting the transition from metaphase to anaphase has been also observed in other Aurora B kinases among other eukaryotes. But the absence from *T. brucei* of the homologs of many of the other essential proteins required in this transition indicates that the mechanisms of metaphase-anaphase transition in *T. brucei* may differ significantly from those in other eukaryotes. We also found that once the anaphase is achieved, the function of TbAUK1 is no longer required for further mitotic progression. But it is needed for the unusual trans-localization of the CPC to the cellular mid-dorsal site in telophase to initiate cytokinesis, albeit unrelated to mitosis. This is the first time to our knowledge that the two functions of an Aurora B homolog have been clearly dissected into two discrete cell cycle stages and separated from each other with little interconnection in between. It may constitute a useful model system for more in-depth understanding of the mechanism of cytokinetic initiation.

## Materials and Methods

### Trypanosome cell culture

The procyclic form of *T. brucei* strain 427 was cultured at 26°C in Cunningham's medium supplemented with 10% fetal bovine serum (Atlanta Biological). Cells were routinely diluted 10-fold whenever the density reached 5×10^5^/ml.

### Epitope tagging of endogenous proteins in *T. brucei*

TbAUK1, TbCPC1 and TbCPC2 were each cloned into the pC-EYFP-Neo vector, which was obtained by replacing the PTP module in the pC-PTP-Neo [43] with the enhanced yellow fluorescence protein (EYFP), and transfected into the wild-type 427 cell line. Correct *in situ* tagging of one of the two alleles was confirmed by PCR and subsequent sequencing. Stable transfectants were selected under 40 µg/ml G418 and cloned by limiting dilution.

### Synchronization of the procyclic form of *T. brucei* and treatment with VX-680

Procyclic cells expressing endogenously tagged TbCPC1-EYFP, TbCPC2-EYFP or TbAUK1-EYFP were synchronized according to the previously described procedure [28] with minor modifications. Instead of using 0.2 mM hydroxyurea to achieve synchronization, 0.3 mM hydroxyurea was added to the cell culture and incubated at 26°C for 16 hrs. Hydroxyurea was washed off with fresh medium and the cells were cultured in fresh medium at 26°C for 8 hrs. VX-680 (30 µM), dissolved in DMSO, was added to the synchronized cells at different time intervals after the release. Time samples of the cell culture were collected by centrifugation, fixed with 4% paraformaldehyde, mounted in the VectaShield mounting medium containing DAPI, and examined for nuclei and kinetoplasts with a fluorescence microscope.

### Flow cytometry

The FACS analysis of propidium iodide-stained trypanosome cells was carried out as previously described [20]. Briefly, *T. brucei* cells were spun down at 320 g for 10 min, washed once in PBS, and re-suspended in 0.1 ml PBS. The cells were fixed by adding 0.2 ml 10% ethanol, 0.2 ml 50% ethanol and 1.0 ml 70% ethanol (all in PBS with 5% glycerol) and incubated at 4°C. The cells were spun down again at 2,900×g for 10 min, washed once with PBS, and re-suspended in PBS. DNase-free RNase (10 µg/ml) and propidium iodide (20 µg/ml) were added before flow cytometry analysis. The DNA content of propidium iodide-stained cells was analyzed with a fluorescence-activated cell sorting scan (FACScan) analytical flow cytometer (BD Biosciences). Percentages of cells in each phase of the cell cycle (G1, S, and G2/M) were determined by the ModFit LT V3.0 software (BD Biosciences).

### Purification of GST fusion protein and *in vitro* kinase assay

Full-length coding sequences of TbAUK1 and histone H3 were each cloned into a pGEM-4T-3 vector (Amersham), expressed in *E. coli* BL21 cells and purified through a column of glutathione Sepharose 4B beads. The purified recombinant proteins were incubated in the kinase buffer (10 mM HEPES, pH 7.5, 50 mM NaCl, 10 mM MgCl_2_, 1 mM DTT) containing 1 µCi [γ-^32^P] ATP (Perkin Elmer) at room temperature for 60 min. Reactions were stopped by adding the SDS sampling buffer and boiled for 5 min. Proteins were separated on SDS-PAGE and the dried gel was exposed to Phosphor-Imager. Equal loading of protein samples in the reaction was verified by Coomassie blue staining of a duplicate SDS-PAGE gel.

### Fluorescence microscopy

Procyclic cells expressing endogenously tagged TbCPC1-EYFP, TbCPC2-EYFP or TbAUK1-EYFP were harvested by centrifugation at 320×g for 5 min, washed once in PBS, and fixed in 4% paraformaldehyde. The fixed cells were washed with PBS, suspended in PBS and adhered to poly-L-Lysine treated cover-slips. The slides were mounted in VectaShield mounting medium containing DAPI and examined with a fluorescence microscope.

### Time-lapse video microscopy

To follow the trans-localization of TbCPC1-EYFP or TbCPC2-EYFP with time-lapse imaging, a melted 1% low melting point agarose mixture in 1.2 ml of SDM79 medium without phenol red and 5 µl Hoechst 33342 solution (1 mg/ml, Invitrogen) was poured onto the center of a slide-glass and covered with another slide-glass. The top slide-glass was then removed from the agarose pad 10–15 min later and the slurry of the transfected cells was poured onto the pad, covered with a cover-slip, and sealed with paraffin. Time-lapse images of individual cells were acquired with a 6D High Throughput Microscope at the Nikon Imaging Center of UCSF. The images were taken at one point with a fixed time interval (1 or 2 min). An auto-focusing program using DIC images was installed that produces images with Z-stacks at −1, 0 and +1 µm from the auto-focused plane. For VX-680 treatment, the drug was added to the culture medium at a final concentration of 30 µM before making the agarose pad. Image acquisition was started about 30 min after pouring the cells on the agarose pad.

## Supporting Information

Figure S1 Effects of VX-680 on cell cycle progression in hydroxyurea-synchronized *T. brucei* procyclic cells. Data from Figure 2 were analyzed with ModFit LT V3.0 software for percentages of cells in G1, S, and G2/M phase in each cell sample.
(1.11 MB TIF)

Figure S2 The time-dependent changes in localization of TbCPC1-EYFP in cells released from hydroxyurea. Cells expressing TbCPC1-EYFP were synchronized with 0.3 mM hydroxyurea for 16 hours, released, harvested every hour, fixed with paraformaldehyde, stained with DAPI and examined under a fluorescence microscope. The arrows point to the spindle structure shown in phase contrast images of the cell.
(7.12 MB TIF)

Figure S3 The time-dependent changes of localization of TbCPC1-EYFP in cells released from hydroxyurea and treated with VX-680 at 0 hr. The time-dependent changes of localization of TbCPC1-EYFP in cells released from hydroxyurea and treated with VX-680 at 0 hr. Cells expressing TbCPC1-EYFP were synchronized with 0.3 mM hydroxyurea for 16 hours, released, treated with 30 µM VX-680 immediately and incubated for 8 hours. Cells were harvested every hour, fixed with paraformaldehyde, stained with DAPI and examined with a fluorescence microscope.
(6.62 MB TIF)

Figure S4 The time-dependent changes of localization of TbCPC1-EYFP in cells released from hydroxyurea and treated with VX-680 1 hr later. See legend of Figure S3.
(6.21 MB TIF)

Figure S5 The time-dependent changes of localization of TbCPC1-EYFP in cells released from hydroxyurea and treated with VX-680 2 hr later. See legend of Figure S3.
(5.01 MB TIF)

Figure S6 The time-dependent changes of localization of TbCPC1-EYFP in cells released from hydroxyurea and treated with VX-680 3 hr later. See legend of Figure S3.
(3.72 MB TIF)

Figure S7 The time-dependent changes of localization of TbCPC1-EYFP in cells released from hydroxyurea and treated with VX-680 4 hr later. See legend of Figure S3.
(3.60 MB TIF)

Figure S8 Effect of VX-680 treatment on size and shape of nucleus. *T. brucei* procyclic cells expressing TbCPC1-EYFP were synchronized with 0.3 mM hydroxyurea, released and treated with 30 µM VX-680 after 0, 1, or 2 hrs. Incubation was continued until the 8th hour after release. Cell samples were fixed in paraformaldehyde, stained with DAPI, and examined with a fluorescence microscope. The control and VX-680-treated cells remained in metaphase with TbCPC1-EYFP concentrated on the metaphase plate. The arrows point to the TbCPC1-EYFP signal concentrated on the metaphase plate, and arrowheads point to the irregularly shaped DAPI-stained DNA.
(6.16 MB TIF)

Video S1 The time course of CPC trans-localization in a *T. brucei* procyclic cell during anaphase to cytokinesis transition. A procyclic cell expressing TbCPC1-EYFP (green) was imaged during anaphase to cytokinesis transition. Time-lapse images were acquired with a fixed time interval (1 or 2 min) with a 6D High Throughput Microscope at the Nikon Imaging Center of UCSF (http://nic.ucsf.edu/6D.html). An auto-focusing program using DIC images was installed that produces images with Z-stacks at −1, 0 and +1 µm from the auto-focused plane. DNA was stained with Hoechst DNA dye (red), and the fluorescence and phase images were merged.
(5.63 MB MOV)

Video S2 Effect of VX-680 treatment of a *T. brucei* procyclic cell prior to the metaphase on CPC trans-localization. A procyclic cell expressing TbCPC2-EYFP (green) was treated with VX-680 when it was released from hydroxyurea treatment and imaged during the following 8 hrs. The cell developed to the metaphase with TbCPC2-EYFP localized to the metaphase plate within the first 2 hrs and remained unchanged for the rest of the time. Details of time-lapse imaging were described in Video S1.
(3.96 MB MOV)

Video S3 Effect of VX-680 treatment of a *T. brucei* procyclic cell during anaphase on CPC translocalization. A procyclic cell expressing TbCPC2-EYFP (green) was treated with VX-680 during anaphase and imaged thereafter. CPC failed to move from the midzone to the mid-dorsal site of the cell and redistributed to the two newly formed nuclei soon thereafter. Details of time-lapse imaging were described in Video S1.
(3.93 MB MOV)
