# Supplementary figures and images for: The Aurora Kinase in Trypanosoma brucei Plays Distinctive Roles in Metaphase-Anaphase Transition and Cytokinetic Initiation
Source: PLoS Pathog. 2009 Sep 11;5(9):e1000575. doi: 10.1371/journal.ppat.1000575 (PMC2734176; doi:10.1371/journal.ppat.1000575)

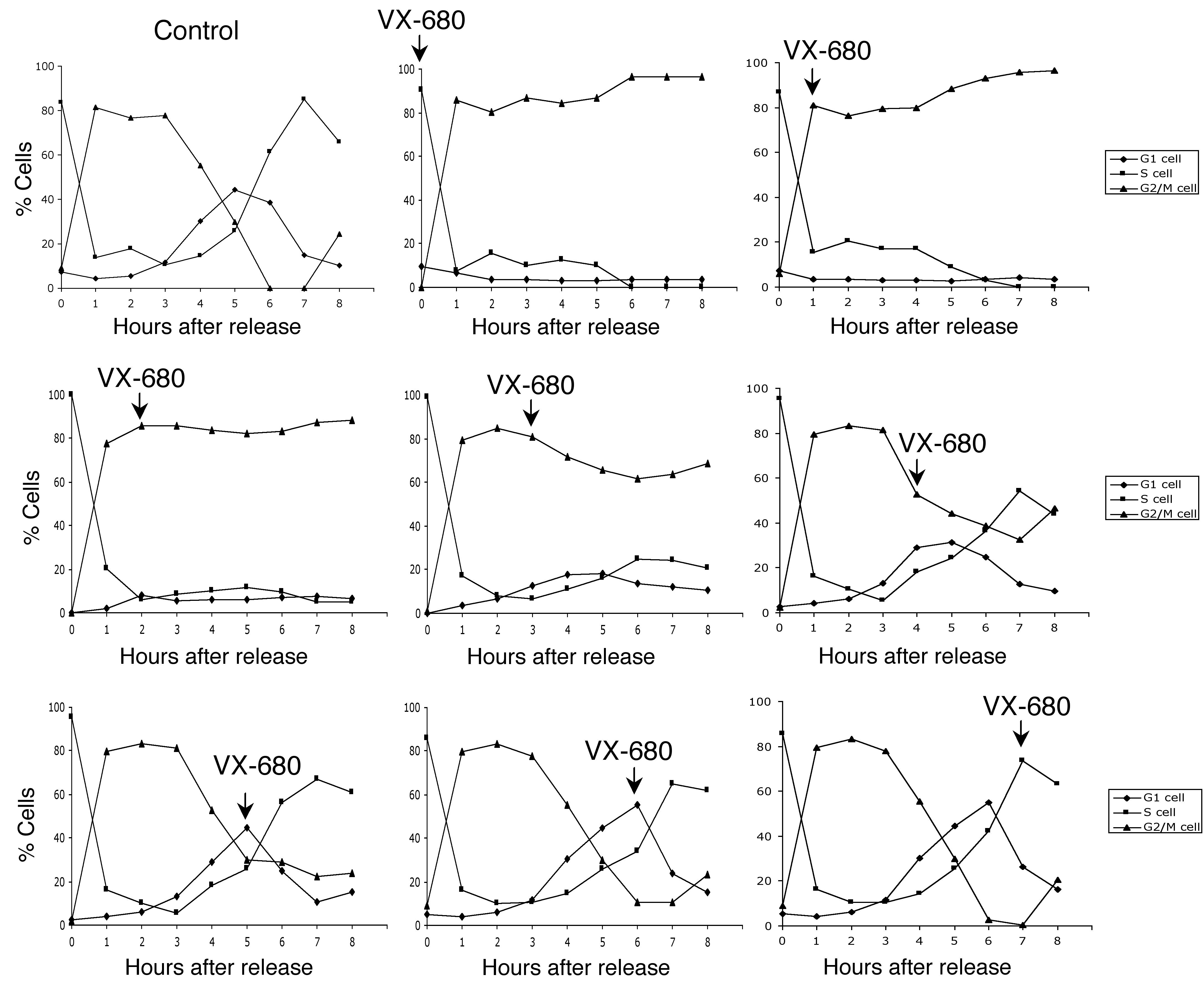

Supplement: Figure S1 — Effects of VX-680 on cell cycle progression in hydroxyurea-synchronized T. brucei procyclic cells. Data from Figure 2 were analyzed with ModFit LT V3.0 software for percentages of cells in G1, S, and G2/M phase in each cell sample. (1.11 MB TIF) [file ppat.1000575.s001.tif]

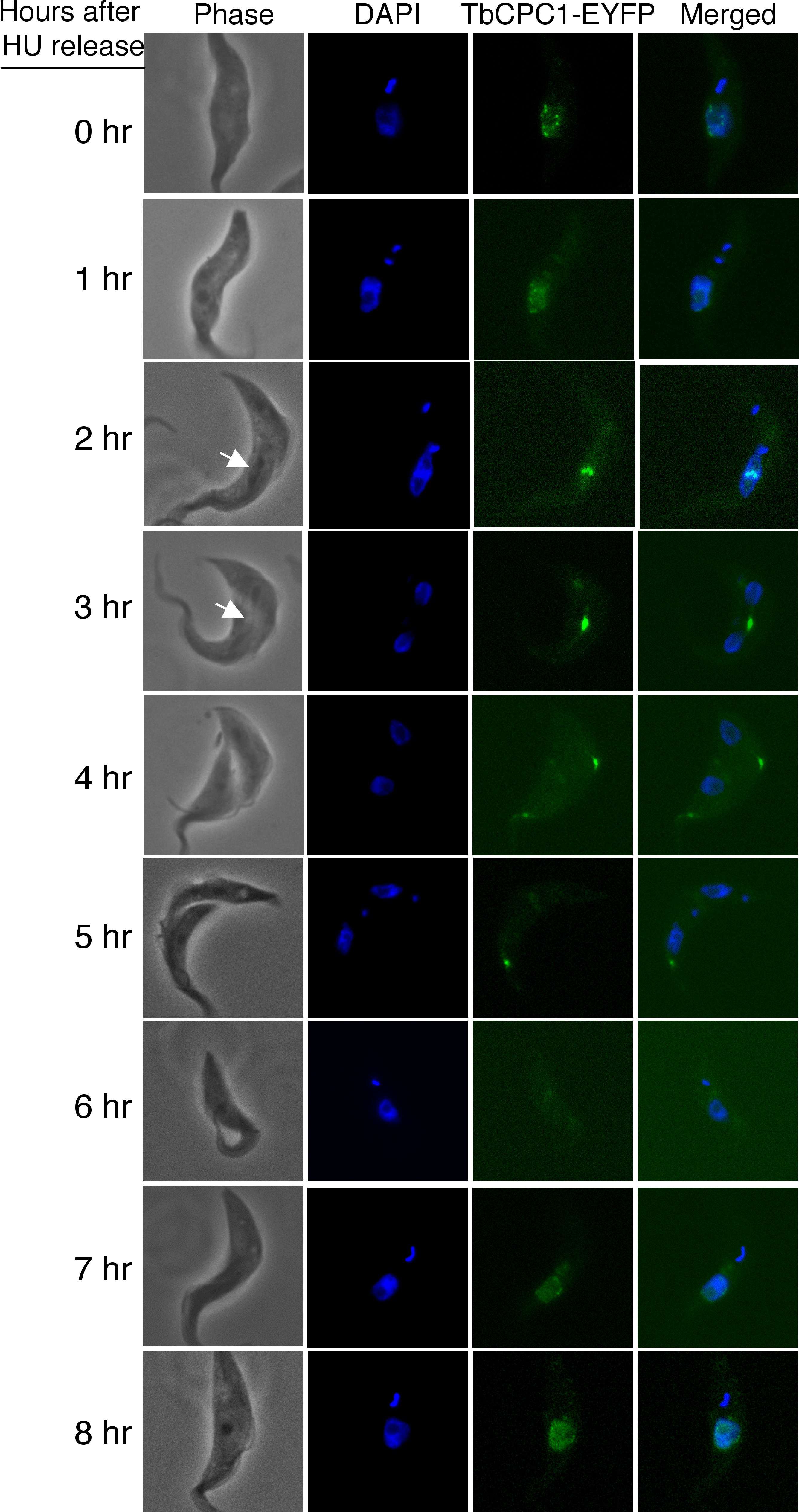

Supplement: Figure S2 — The time-dependent changes in localization of TbCPC1-EYFP in cells released from hydroxyurea. Cells expressing TbCPC1-EYFP were synchronized with 0.3 mM hydroxyurea for 16 hours, released, harvested every hour, fixed with paraformaldehyde, stained with DAPI and examined under a fluorescence microscope. The arrows point to the spindle structure shown in phase contrast images of the cell. (7.12 MB TIF) [file ppat.1000575.s002.tif]

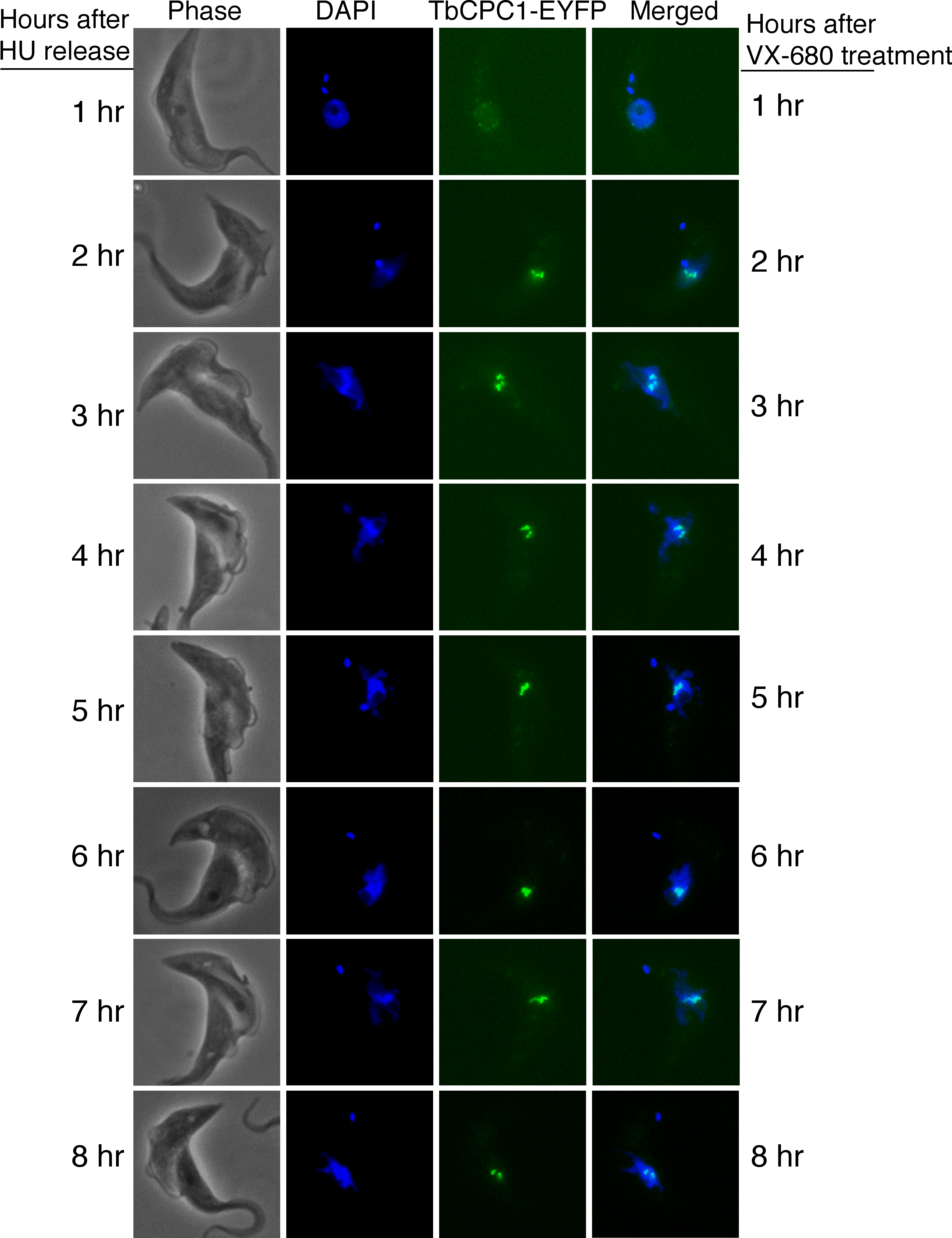

Supplement: Figure S3 — The time-dependent changes of localization of TbCPC1-EYFP in cells released from hydroxyurea and treated with VX-680 at 0 hr. The time-dependent changes of localization of TbCPC1-EYFP in cells released from hydroxyurea and treated with VX-680 at 0 hr. Cells expressing TbCPC1-EYFP were synchronized with 0.3 mM hydroxyurea for 16 hours, released, treated with 30 µM VX-680 immediately and incubated for 8 hours. Cells were harvested every hour, fixed with paraformaldehyde, stained with DAPI and examined with a fluorescence microscope. (6.62 MB TIF) [file ppat.1000575.s003.tif]

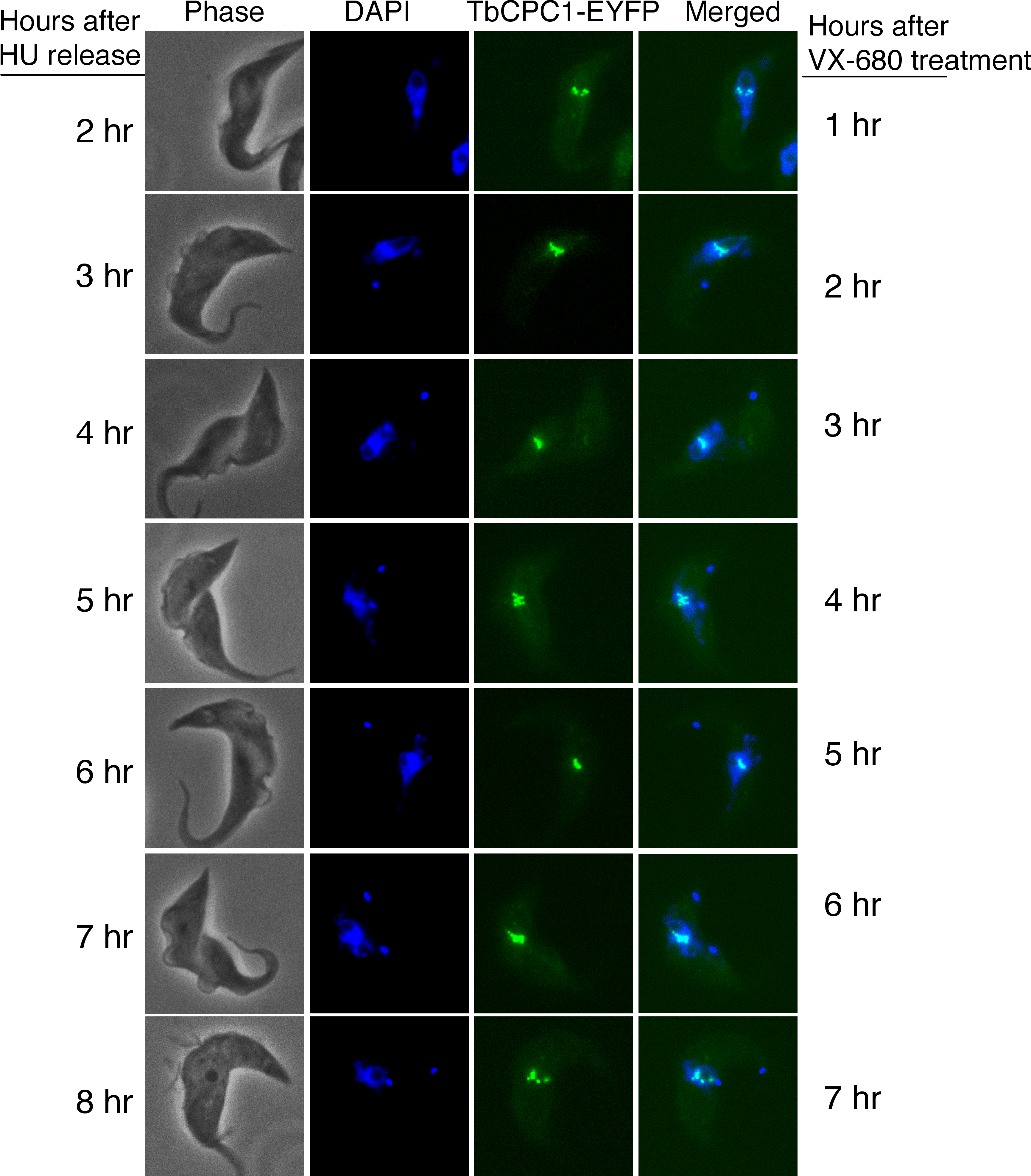

Supplement: Figure S4 — The time-dependent changes of localization of TbCPC1-EYFP in cells released from hydroxyurea and treated with VX-680 1 hr later. See legend of Figure S3. (6.21 MB TIF) [file ppat.1000575.s004.tif]

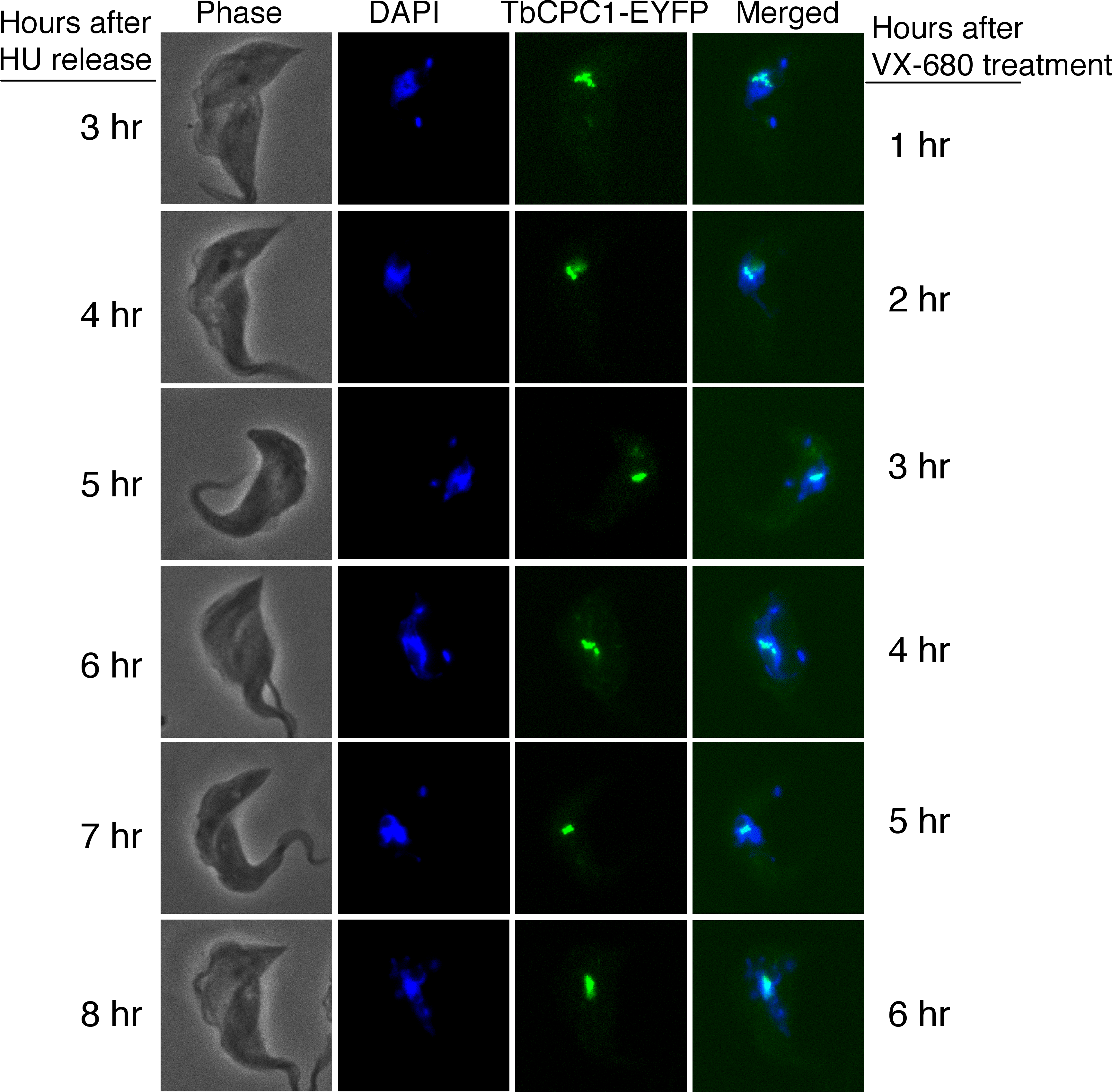

Supplement: Figure S5 — The time-dependent changes of localization of TbCPC1-EYFP in cells released from hydroxyurea and treated with VX-680 2 hr later. See legend of Figure S3. (5.01 MB TIF) [file ppat.1000575.s005.tif]

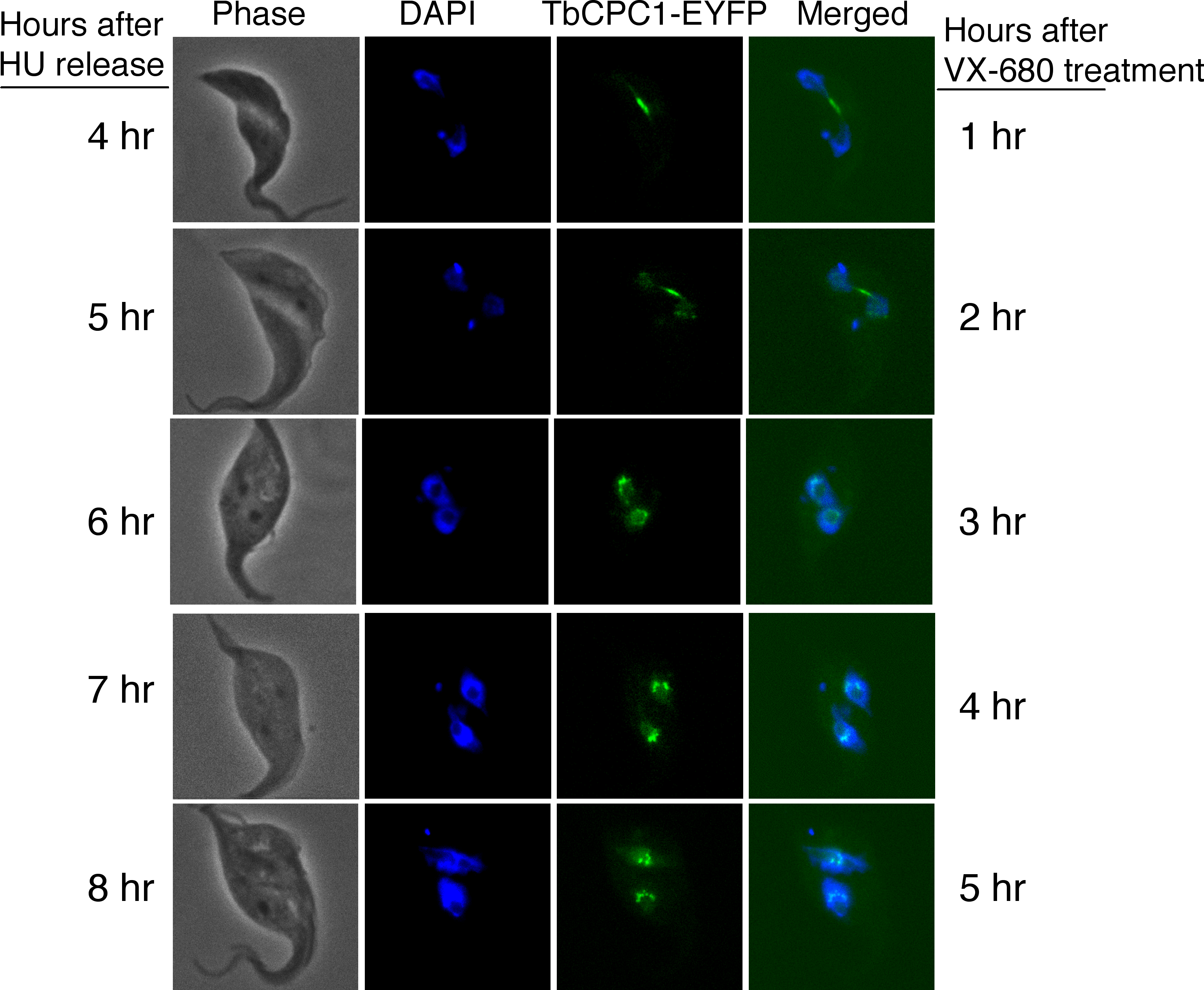

Supplement: Figure S6 — The time-dependent changes of localization of TbCPC1-EYFP in cells released from hydroxyurea and treated with VX-680 3 hr later. See legend of Figure S3. (3.72 MB TIF) [file ppat.1000575.s006.tif]

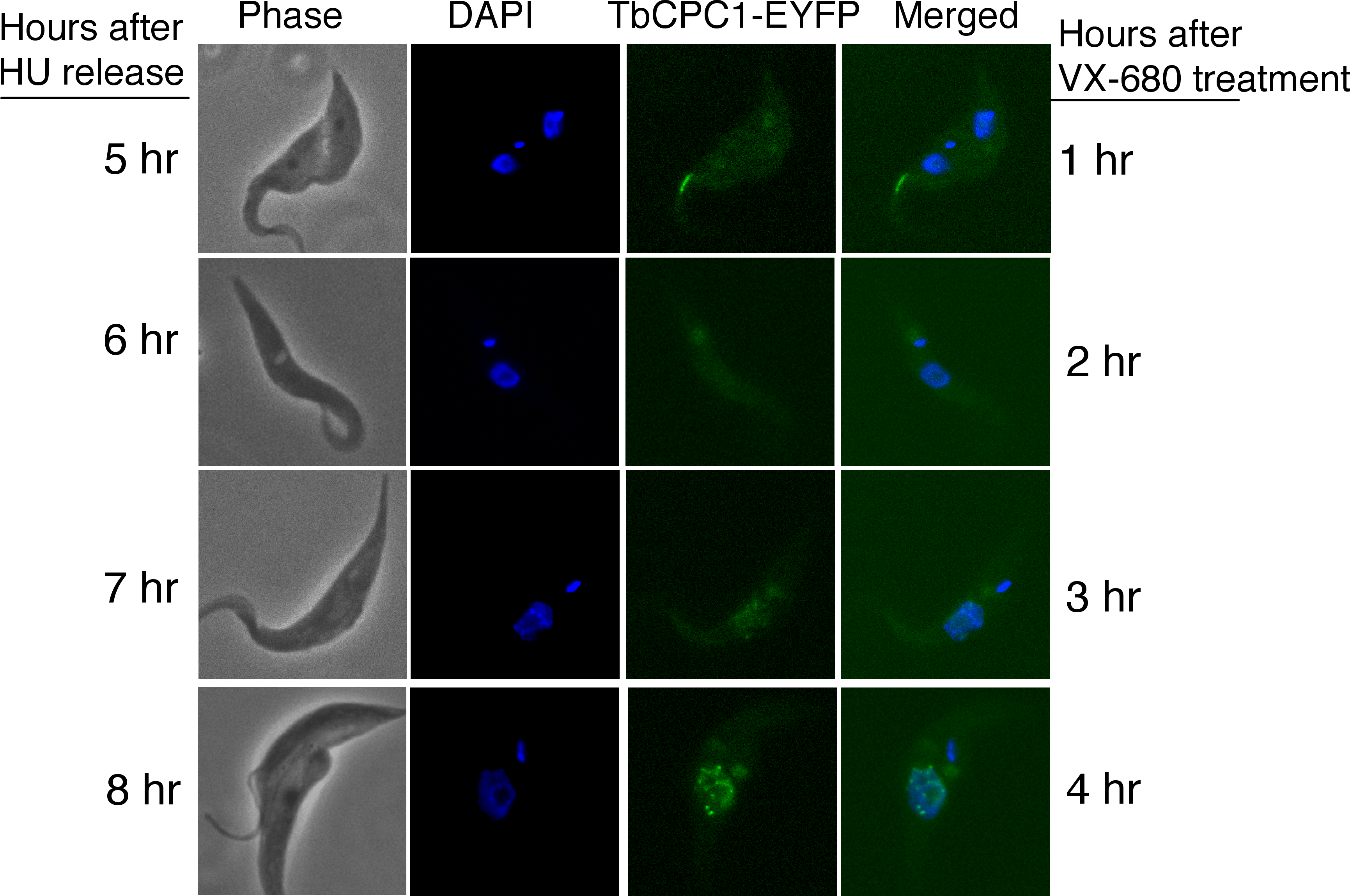

Supplement: Figure S7 — The time-dependent changes of localization of TbCPC1-EYFP in cells released from hydroxyurea and treated with VX-680 4 hr later. See legend of Figure S3. (3.60 MB TIF) [file ppat.1000575.s007.tif]

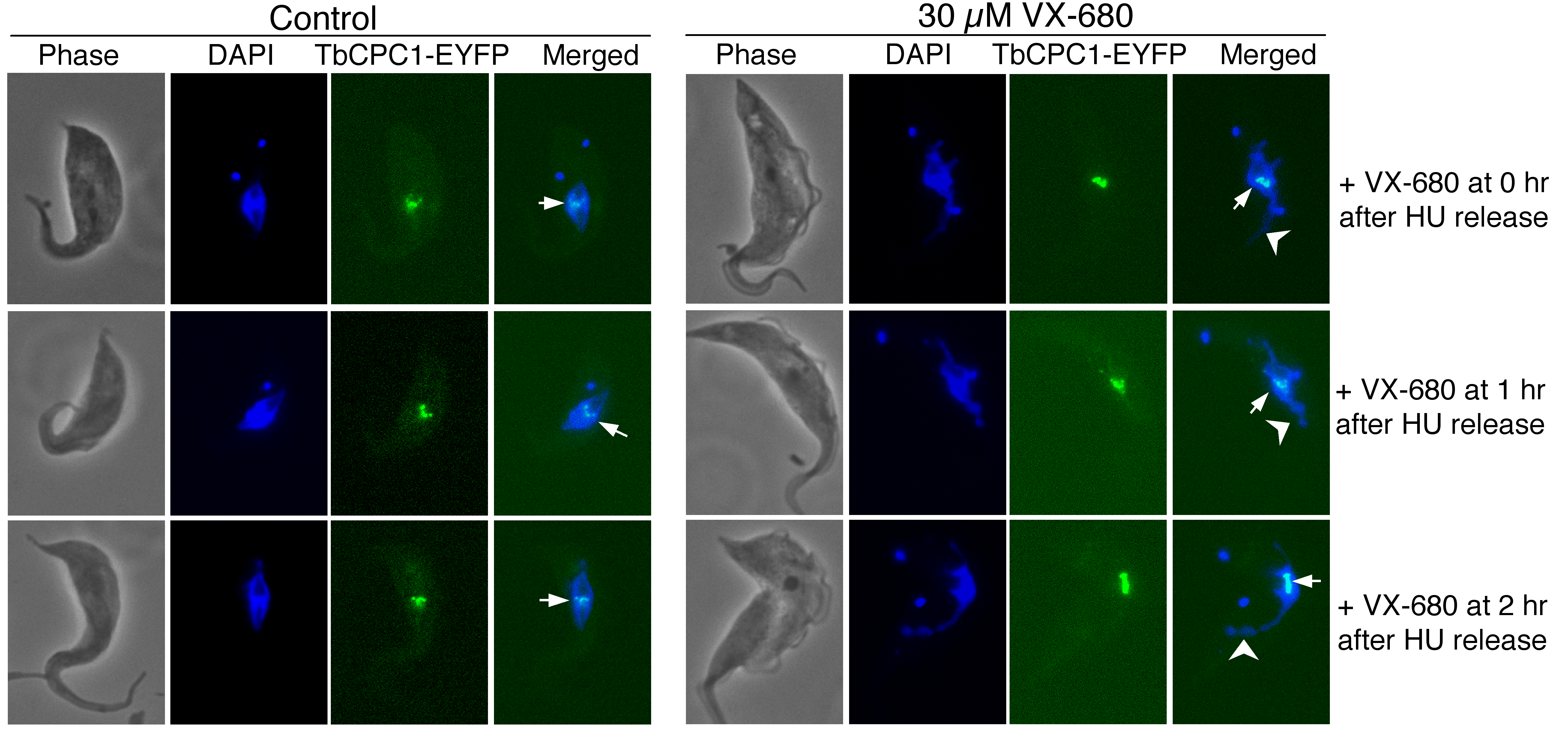

Supplement: Figure S8 — Effect of VX-680 treatment on size and shape of nucleus. T. brucei procyclic cells expressing TbCPC1-EYFP were synchronized with 0.3 mM hydroxyurea, released and treated with 30 µM VX-680 after 0, 1, or 2 hrs. Incubation was continued until the 8th hour after release. Cell samples were fixed in paraformaldehyde, stained with DAPI, and examined with a fluorescence microscope. The control and VX-680-treated cells remained in metaphase with TbCPC1-EYFP concentrated on the metaphase plate. The arrows point to the TbCPC1-EYFP signal concentrated on the metaphase plate, and arrowheads point to the irregularly shaped DAPI-stained DNA. (6.16 MB TIF) [file ppat.1000575.s008.tif]
